# Ongoing egocentric spatial processing during learning of non-spatial information results in temporal-parietal activity during retrieval

**DOI:** 10.3389/fpsyg.2013.00366

**Published:** 2013-06-25

**Authors:** Alice Gomez, Mélanie Cerles, Stéphane Rousset, Jean-François Le Bas, Monica Baciu

**Affiliations:** ^1^Laboratoire de Psychologie et NeuroCognition, CNRS, UMR 5105, University of Grenoble AlpesGrenoble, France; ^2^Grenoble Institut des Neurosciences, Unité IRM, IFR1, CHU GrenobleGrenoble, France

**Keywords:** allocentric, navigation, episodic memory, spatial, parieto-temporal pathway, precuneus

## Abstract

Deficits in amnesic patients suggest that spatial cognition and episodic memory are intimately related. Among the different types of spatial processing, the allocentric, relying on the hippocampal formation, and the egocentric-updated, relying on parieto-temporal connections have both been considered to functionally underlie episodic memory encoding and retrieval. We explore the cerebral correlates underlying the episodic retrieval of words previously learnt outside the magnet while performing different spatial processes, allocentric and egocentric-updated. Subsequently and during fMRI, participants performed an episodic word recognition task. Data processing revealed that the correct recognition of words learnt in egocentric-updated condition enhanced activity of the medial and lateral parietal, as well as temporal cortices. No additional regions were activated in the present study by retrieving words learnt in allocentric condition. This study sheds new light on the functional links between episodic memory and spatial processing: The temporo-parietal network is confirmed to be crucial in episodic memory in healthy participants and could be linked to the egocentric-updated process.

## Introduction

Episodic memory was originally described as the ability to recollect specific events and includes spatial and temporal information of the individual's life (Tulving, 1972). Based on this original definition and on neuropsychological findings in amnesic patients, it is generally agreed that spatial cognition and episodic memory are intimately linked (O'Keefe and Dostrovsky, 1971; O'Keefe and Nadel, 1978; Holdstock et al., 2000; Spiers et al., 2001a,b; Burgess et al., 2002; King et al., 2002).

According to the Cognitive Map theory (O'Keefe and Nadel, 1978), allocentric spatial maps stored in the hippocampus have evolved in humans to support the spatio–temporal context of episodic memories (Burgess, 2008). Indeed, allocentric representations, which are independent of subject movement, would be better suited than other representations to support long-term memory storage (Burgess et al., 2001a,b). The hippocampus provides a spatial scaffold for the episode, binding all the neocortical representations related to an episode. Thus, the Cognitive Map Theory suggests that the link between episodic memory encoding and retrieval, and allocentric representations mainly involves hippocampal regions.

On the basis of purely spatial constraints, Byrne, Becker, and Burgess (Becker and Burgess, 2001; Burgess et al., 2001a; Byrne et al., 2007) proposed a computational model of the neural mechanisms that underlie spatial memory and imagery. Based on a neurofunctional model of spatial memory, its extension to memory for contexts provided a formal model of the role of spatial cells described in the Cognitive Map Theory (O'Keefe and Nadel, 1978) and additionally involved parietal-temporal areas in egocentric spatial processing (Burgess et al., 2001a). This computational model was extended to explain event memory: during encoding, an event is initially perceived in an egocentric-parietal reference frame (i.e., static self-to-object relationship, like retinotopic snapshots) and is then translated through the egocentric-updating process (dynamic self-to-object relationship, mediated by extracting clues from interaction with the environment through self-motion; Burgess, 2008) into an allocentric-hippocampal reference frame. Upon retrieval, an allocentric trace is reactivated and translated back into an egocentric reference frame providing a specific perspective of the event. Interestingly, this translation mechanism explains unaccounted aspects of the phenomenology of episodic retrieval. It is accepted that episodic memory preferentially refers to autonoetic consciousness, the ability to project oneself in the past (Wheeler et al., 1997; Tulving, 2002). Beyond the phenomenological state of autonoetic consciousness described as centered on the self, behavioral studies have shown that the retrieval of episodic autobiographical events is more frequently associated with a first-person perspective than semantic autobiographic descriptions (Crawley and French, 2005; Eich et al., 2009).

Extending the Byrne et al. model (2007), the role of the egocentric perspective during retrieval has then been suggested to be critical to autonoetic consciousness in reexperiencing from a first-person perspective during episodic retrieval (Rosenbaum et al., 2004; Gomez et al., 2009, 2012; Ciaramelli et al., 2010; Hirshhorn et al., 2012). For instance, Ciaramelli et al. (2010) have shown that patients suffering from posterior parietal lesions are unable to retrieve remote spatial memories within an egocentric framework. If this observation is not fully conclusive for the posterior parietal involvement in episodic memory, it suggests a role of posterior parietal regions in recreating an egocentric perspective during episodic retrieval (see also e.g., Wagner et al., 2005; Cabeza, 2008 for alternative views on the parietal involvement in episodic memory). The posterior parietal cortex has also been involved in recollective experience in brain-lesioned patients (Berryhill et al., 2007; Simons et al., 2008; Davidson et al., 2010).

To formalize the functional processing at hand, some research focusing on the reexperiencing of episodic memories during retrieval have proposed to adapt the Burgess, Becker and Byrne model by adding a memorization step to the initial transformation process (Gomez et al., 2009, 2012; Serino and Riva, 2013). The translation process would rely on egocentric-updating spatial processing. Centered on the observer, egocentric-updated processes dynamically codes for self-to-environment relations during navigation, using vestibular, proprioceptive, and visual continuous inputs (Farrell and Robertson, 1998). Therefore, it promotes an immersive sense of space as well as the agency of the self in action. Hence, during the encoding of any event, the initial transformation from egocentric to allocentric is memorized by the system. During retrieval of this event, the memory of the transformation mechanism leads to a fluency in reinstating a specific egocentric perspective. This fluency is responsible for the feeling of autonoetic consciousness (see e.g., Jacoby et al., 1989; Whittlesea, 1993, for a link between fluency and memory indicators in the perceptual and conceptual domains). This memorization step is not included in the BBB model in which the translation process does not trigger consciousness mechanisms at retrieval. This memory mechanism would allow individuals to distinguish imagination built on semantic knowledge from recollecting true experiences. In brief, memorizing the translation process would play a decisive role in the access to a state of autonoetic consciousness (Gomez et al., 2009; Cerles and Rousset, 2012). Neuropsychological evidence supporting this view have related egocentric-updated deficits to episodic memory deficits in an amnesic patient (Gomez et al., 2012). This patient, who suffered from bi-hippocampal amnesia, a specific deficit of episodic memory, also showed a deficit in spatial processing restricted to the egocentric-updated process. However, this case-report did not clearly point to a specific neural substrate involved in such a functional link between spatial processing and episodic memory, because the temporal lesion extended to parietal areas.

Hence models linking space and episodic memory have supposed that allocentric and egocentric-updated processings of space are involved during the encoding and the retrieval of episodic memory.

fMRI studies linking spatial processes to memory have described the cerebral substrate involved by different spatial perspective during encoding (Shelton and McNamara, 2004a,b; Wolbers and Büchel, 2005) or during the retrieval of spatial informations from memory (Maguire et al., 1997; Parslow et al., 2004; Rosenbaum et al., 2004, 2007; Hoscheidt et al., 2010). In general, egocentric spatial retrieval involves the dorso-parietal areas, whereas allocentric spatial retrieval involves the temporal regions and in particular the hippocampus. For instance, Hoscheidt and colleagues compared hippocampal activation during spatial and non-spatial relational judgments in semantic and episodic versions of the same task. Among other results, they reported that spatial relational judgments always elicited greater hippocampal activation compared to non-spatial judgments independently of their semantic or episodic nature. This finding supports the view that the hippocampus contributes to retrieval when space and spatial relations are voluntarily invoked by the participants. However, no fMRI studies assessed whether the retrieval of a non-spatial element from episodic memory automatically involved spatial processes. Nevertheless, all the models previously described clearly predict that such retrieval should automatically invoke spatial processes and they also make distinct predictions on the type of spatial processes involved (egocentric-updated and allocentric).

The goal of the study was to understand how the spatial processing as a contextual component of encoding affects episodic memory retrieval as an automatic process (i.e., with no artificial additional verbal command to trigger spatial processing upon retrieval) at a cerebral level. Because the distinction in terms of spatial processes has been previously hypothesized to be important for models of episodic memory, we tested the effect of two spatial processes performed during learning, an allocentric and an egocentric-updated. Surprisingly, although this distinction in terms of spatial processing is hypothesized to be important for models of episodic memory, their automatic effect during the retrieval of a non-spatial element is still unknown.

According to the Burgess et al. model (e.g., Burgess et al., 2001b) and the Gomez et al. (2009, 2012), which convey a strong role of self-perspective in retrieval, specific activity should be observed in the egocentric-updating condition, in regions supporting this process, such as the parieto-temporal pathway for instance. On the other hand, the Cognitive map and the BBB model predict a specific role of allocentric processing for encoding and retrieval in episodic memory. Because all theories predict that the hippocampi are involved in spatially binding elements of the episode during retrieval, an involvement of the hippocampal formation during retrieval can be expected.

## Materials and methods

### Participants

Twenty adults (age range = 17–30, mean age = 23.5, *SD* = 2.5, 13 males) took part in the experiment. All participants were right-handed according to Edinburgh Handedness Inventory (Oldfield, 1971). They gave their informed written consent for the experiment and the study was approved by the local ethics committee (CPP n°08-CHUG-10, 20/05/2008).

### Procedure overview

The experimental procedure consists of two phases: (1) *Spatial tasks with word learning* and (2) *Word recognition*. Word learning was performed outside the magnet and word recognition inside the magnet. In the learning phase, participants had to perform the spatial tasks (allocentric or egocentric-updated) while learning the words. Six-hours later, the episodic word recognition was performed inside the magnet.

### Outside MR magnet: spatial tasks and word learning

#### Stimuli

The trials were displayed on a computer monitor using E-prime software (E-prime Psychology Software Tools Inc., Pittsburgh, USA) for learning the words. Thirty-six trials were presented (18 in egocentric-updated condition, 18 in allocentric condition). Each word-to-be-learnt was selected from the plant category, as well as the 36 additional filler words used in the recognition phase. Overall, these words had a lexical frequency use of 2.62 occurrences per a million (*SD* = 2.69) according to the word frequency database Lexique 3.55 (New et al., 2001, 2004, http://www.lexique.org). Both lists were counterbalanced across spatial conditions.

**Figure 1:**
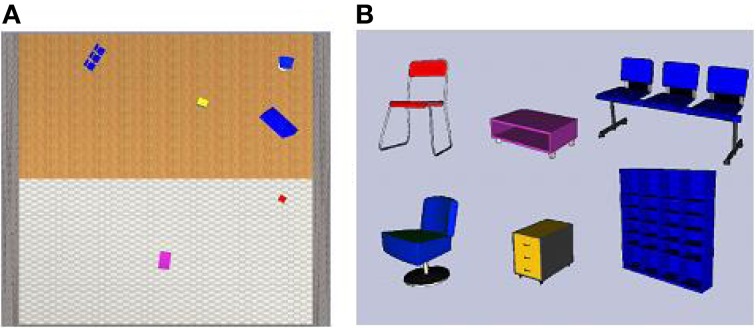
**(A)** Map-view of the 9 × 9 m room (stone walls, tile and wooden flooring), with a particular spatial configuration of six objects presented during the learning phase **(B)** side-view of six objects used to compose the spatial environment for all the contextual spatial tasks. Each object picture was used separately to create the target pictures used during the contextual spatial tasks.

Concerning the spatial tasks, the layout configuration presented in each film was always different and contained an average of five objects ranging from 4 to 6 objects. Each scene layout was used once for each condition, resulting in 36 spatial films of 18 s each for the task (20 additional spatial films were made to train participants beforehand, See Figure 1).

Egocentric-updated films presented a straight view from the perspective of a 180 cm tall observer; camera movement made it possible to simulate the view of an observer walking through the environment to enhance both spatial immersion and the sense of self agency (see Video 1, Gomez et al., 2013a, http://figshare.com/articles/Egocentric_updating_video_example/695840). On the contrary, allocentric films showed a bird's eye perspective, looking straight down, with 15% of the environment visible at any moment and the camera scanned the map of the environment with a fixed orientation (see Video 2, Gomez et al., 2013b, http://figshare.com/articles/Allocentric_video_example_Video_2/695839). The camera movement simulated a path of about 10 m long with one or two direction changes, and a speed of a moderately paced walk (1.5 m/s).

**Figure 2:**
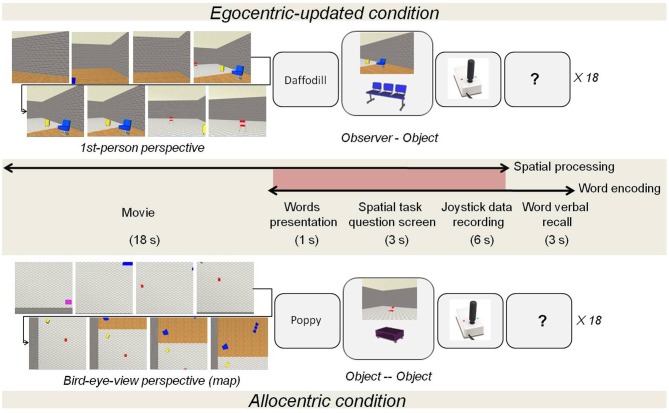
**Learning phase procedure according to the type of spatial processing, egocentric-updated or allocentric**. The participants performed the following successive operations: (a) first watched the film, (b) learnt a word (e.g., daffodil), (c) saw the screen with the spatial task question, (d) answered the question by using a joystick and (e) pronounce overtly the last seen word.

The origin and the object-to-be pointed pictures were selected from the first and second half of the film, respectively (9 s delay minimum) and presented together (see Figure 2).

#### Procedure

During this phase, participants had to carry out two tasks concomitantly (See Figure 2): word learning and spatial task (either egocentric-updated or allocentric). This phase was structured as follows: (A) spatial encoding phase with film presentation (18 s); (B) word presentation (1 s); (C) spatial test (9 s), (D) short-term word recall (3 s) (See Figure 2).

In the spatial task, participants had to memorize the position of objects displayed in the film (either egocentric-updated or allocentric). Egocentric-updated showed a ground-level 1-st person-perspective. Instead, allocentric films showed a survey perspective. During the test, using a joystick, participants used different spatial referencing (i.e., egocentric-updated vs. allocentric) to point in the direction of the presented object. With that aim, two objects were presented for 3 s: Picture 1, the origin of the spatial referencing and Picture 2, the object-to-be-pointed. For the egocentric referencing to occur, participants were asked to immerse themselves in the Picture 1, and to point from their immersed position (i.e., self-to-object pointing). In contrast, for the allocentric referencing to occur participants were instructed to imagine that they were sketching the direction on the map of the environment and to point the direction of an object relative to another object in the fixed referencing of the environment (i.e., object-to-object pointing relative to the fixed orientation of the map). An allocentric centered joystick picture was used to prompt participants' response and to collect the behavioral performance.

During the word learning task, participants were instructed to retain the word that was presented in each trial, and recall it verbally at the end of each trial. Participants were involved in a dual-task situation period: first, the spatial information from the scene must be kept in mind at the same time as the word (encompassing its own spatial reference, screen location, relation to participant…); then, they had to solve the spatial task during the word short-term memory maintenance. All participants completed the verbal recall with full success. Participants were not aware that they would have to recognize these words afterwards.

Beforehand, all participants were trained to perform the spatial tasks (without word learning) with 10 trials of each condition. During this training, participants were rewarded by a visual feedback on their pointing response to improve performance (from online data recording). Angle errors were recorded on each trial using in-house software (VRML-prime: http://webu2.upmf-grenoble.fr/LPNC/membre_eric_guinet). A control experiment performed on the same participants allowed us to check that both spatial tasks were equivalent in terms of complexity. In this control experiment, the absolute error angle was computed on each trial by comparing the expected angle to the produced angle. Participants performed with an average absolute error-angle size of 35.5° (*SD* = 13.5). No absolute error-angle size difference (*F* < 1) was observed between both spatial task conditions (allocentric and egocentric-updated).

### During scanning: word recognition

#### Procedure

**Figure 3:**
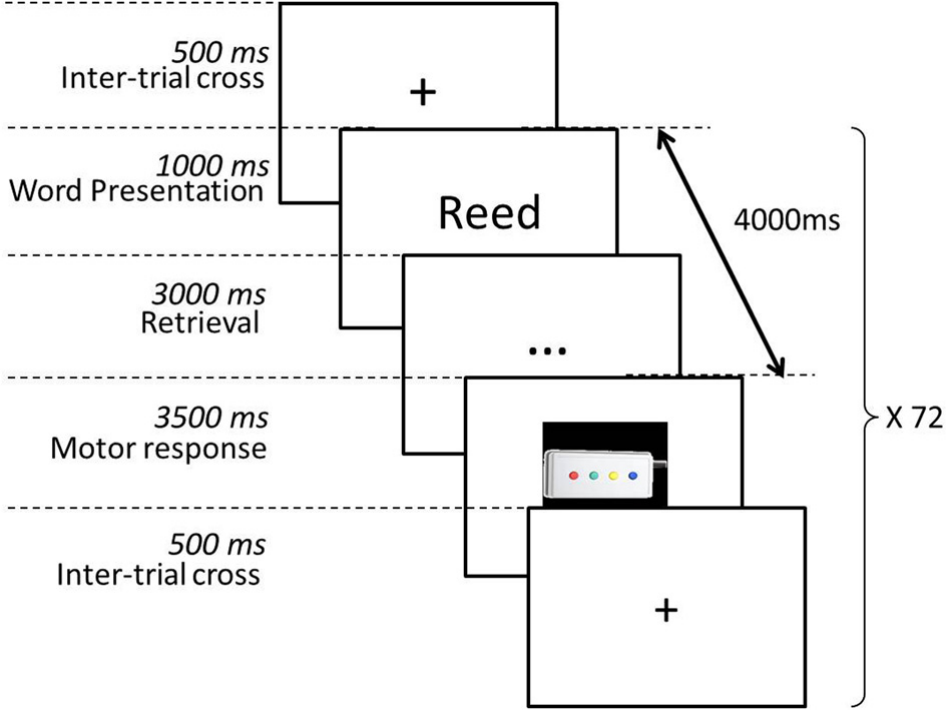
**Word recall phase procedure**. Only new and old words are displayed. Participants are prompted to decide whether the words were previously learnt. Words are classified as hits (words learnt previously correctly recognized), false recognition, misses, or correct rejections. Hit words are separated into two categories: (1) learnt in an egocentric-updated condition or (2) learnt in an allocentric condition.

Six hours after the learning phase, participants carried out an incidental episodic recognition task within an event-related fMRI paradigm (see Figure 3). Participants had to decide whether the word was learnt in the previous phase. Word conditions (allocentric condition, egocentric-updated, and new) were pseudo-randomly interleaved. Each trial displayed a word (1 s), ellipsis dots (3 s), and a response key pictures (3.5 s) to allow participants to respond. Each trial was separated by an inter-trial presenting a cross during 0.5 s. (see Figure 3). They were instructed to press one of four response keys on a manual system: key 1 “I do not recognize the word,” key 2 “I am not sure if I have seen it or not,” key 3 “I do recognize the word,” key 4 “I recognize the word and I remember when I learnt it, I remember some aspects and details of this episode” (Gardiner et al., 1998; Gardiner, 2001). If participants requested supplementary explanations of how they have to perform the task, the experimenter provided examples of associations made with another thought or idea emerging at the same time that they learned the word, but the experimenter was careful not to refer to spatial aspects. Key 1 and 2 were supposed to reflect unrecognized words. Key 3 and 4 were supposed to reflect recognized words. Because a maximum of only 18 words could be recognized, we planned to pool all correct recognitions which were not a simple guess. This procedure was chosen for two reasons: (1) to focus participants' attention on a potential recollection of the event and (2) to lead them to make recognition judgments based on the amount of available evidence (Malmberg, 2008). Before entering into the magnet, participants were first trained to respond timely with irrelevant verbal stimuli. Each participant performed the episodic word recognition task during one scan (run) with 72 stimuli of two types, learnt words (36) and filler words (36).

It's important to note that the use of a response deadline for the decision process was expected to lower the decision criteria used by participants to give overt responses (Yonelinas, 2002) compared with an untimed experiment. In fact, in similar conditions (items from an homogenous category) but without a response deadline, we have observed that numerous recollection decision responses occurred after 8000 ms (Gomez et al., 2009, Recognition duration, *M* = 7400 ms, *SD* = 10200 ms). As such we hoped that using a response deadline, which was not restricted to familiarity processes (i.e., >700 ms, Yonelinas, 2002) but which could prevent participants from recollecting the entire event, would lead us to equivalence across conditions in the success rate, while still engaging participants in the recollective process (as suggested by the high rate of R responses). A previous study (Gomez et al., 2009) showed that when given unlimited time to respond, an egocentric-updated learning phase led to more episodic recall than a allocentric learning phase. If such a difference in the learning rate had occurred it would have been difficult to discard the eventuality that differential activations between conditions where not only driven by a feeling of success in the egocentric-updated condition. In fact, error detection and monitoring of self-performance is known to engage regions from the cingulate cortex (Charles et al., 2013), and regions in the parietal and right frontal areas during successful retrieval (McDermott et al., 2000). As a consequence difference in brain activations associated with differential rate of success across memory conditions are difficult to interpret.

Indeed, as expected the ANOVA conducted on the number of hits did not show any significant effect (*F* < 1) of the type of spatial processes performed during learning (egocentric-updated, *M* = 13.45, *SD* = 2.6, allocentric, *M* = 14, *SD* = 1.9). The behavioral responses were still correct on most trials (*M* = 71.9%, *SD* = 8.4%, including hits and correct rejection) and above chance level (*T* = 11.6, *p* < 0.001). The average d' was significantly different from 0 [d' = 2.78 (1.07), *T*_(19)_ = 11.92, *p* < 0.001] suggesting that participants could accurately distinguish words presented in the learning phase (hits, *M* = 89%, *SD* = 8%) from new words (False alarms, *M* = 14%, *SD* = 10%). Moreover, the overall correct detection scores (hits) were significantly correlated with d' scores (*r* = 0.47, *p* < 0.05). Hence, in the present study, participants who make more hits are also those who are more likely to correctly classify an item as old and new. Moreover, in line with previous studies (Yonelinas, 2002), with a response deadline greater than 700 ms, most responses were associated to a detailed recognition [*F*_(1, 19)_ = 28.63, *MSE* = 24.934, *p* < 0.001, *M* = 19.7, *SD* = 5.9] compared to a simple episodic recognition (*M* = 7.75, *SD* = 4.7). The proportion of detailed recognition (*M* = 70%, *SD* = 21 in allocentric condition, *M* = 72%, *SD* = 21 in egocentric-updating) and simple episodic recognition (*M* = 30%, *SD* = 17 in allocentric, *M* = 28%, *SD* = 17 in egocentric-updating) was also similar in both conditions as reflected by the lack of interaction effect between response types and the spatial processes performed during learning (*F* < 1).

For this functional scan, 200 functional volumes were acquired with an average inter-stimulus interval of 8 s. The duration of the functional scan was 10 min.

#### MR acquisition and data processing

Magnetic resonance scanning was carried out on a 3T MRI Scanner (Bruker MedSpec S300) with a standard head coil. We acquired 39 axial slices (slice thickness, 3.5 mm) using a gradient gradient-echo/T2^*^ weighted EPI method (matrix, 72 × 72; field of view, 216 × 216 mm). The main sequence parameters were: TR = 3 s, TE = 30 ms, flip angle = 77°. The TR was thus asynchronous with the SOA resulting in an effective sampling rate of the BOLD response. An LCD projector back-projected the virtual environment on a screen positioned behind the head coil. Participants lay on their backs in the bore of the magnet and viewed the stimuli binocularly via a 45° mirror which reflected the images displayed on the screen. To minimize head movements, participants were stabilized with tightly packed foam padding surrounding the head.

Image processing and statistical analysis of fMRI data were carried out using SPM5 (Welcome Department of Imaging Neuroscience, London, UK, www.fil.ion.ucl.ac.uk/spm). All volumes were realigned to the reference volume, spatially normalized to T1-weighted anatomical volume in a standard coordinate system and finally smoothed using a 8-mm full-width at half-maximum isotropic Gaussian kernel. Time series for each voxel were high-pass filtered (1/128 Hz cutoff) to remove low frequency noise and signal drift. After spatial pre-processing steps, the statistical analysis was performed separately, on the functional images acquired for each task.

Words were defined by several factors: spatial processing during learning (allocentric vs. egocentric-updated, only for old words), word status (old vs. new) and *a posteriori*, participants response (recognized vs. rejected). This resulted in 6 experimental conditions declared as separate factors in the fMRI analysis: allocentric words correctly recognized (allocentric hits), egocentric-updated words correctly recognized (egocentric-updated hits), allocentric words rejected (allocentric misses), egocentric-updated words rejected (egocentric-updated misses), new words recognized (false alarms), and new words rejected (correct rejections). This resulted in an average of 13.7 events (*SD* = 2.2) in the allocentric hits and egocentric-updated hits condition and an average of 24.35 events (*SD* = 5.7) in the correct rejections condition.

The conditions of interest (allocentric recognized/egocentric-updated recognized/new rejected) were modeled as three regressors convolved with a canonical hemodynamic response function (HRF). The movement parameters derived from the realignment corrections (three translations and three rotations) were also entered in the design matrix as additional factors. The general linear model was then used to generate the parameter estimates of the activity for each voxel, each condition and each participant. Statistical parametric maps were generated from linear contrasts between the HRF parameter estimates for the different experimental conditions. An approximate AR(1) autocorrelation model estimated at omnibus *F*-significant voxels (*p* < 0.05, FDR corrected for hits vs. correct rejection, *p* < 0.0001 uncorrected for spatial condition contrasts) was used globally over the whole brain.

Our main goal was to identify the cerebral regions whose activity during correct word recognition (Hits) was driven by the spatial condition, by contrasting egocentric-updated hits with allocentric hits and vice versa. Specific effects of spatial processes performed during learning were tested with appropriate linear contrasts (i.e., egocentric-updated hits vs. allocentric hits and allocentric hits vs. egocentric-updated hits) of the parameter estimates. The corresponding contrast images were subsequently entered into a random effects group analysis.

After examining the contrasts specific to each spatial processing, we turned to the specific areas for memory processes. We first contrasted hits vs. new words rejected to replicate previous data on the memory network. However, we tried to specify the neural substrate activated by word retrieval during an episodic recognition task. More specifically, we investigated how between-subjects variability may be related to BOLD responses and which are the regions of this recognition network modulated by this performance variability (via a correlation between BOLD response and correct detection scores). In order to answer this question, we included the individual contrast images of mean activation during retrieval (egocentric-updated and allocentric hits vs. Correct rejection, one image per participant) and each participants' average correct word detection score served as a predictor variable in multiple regression analysis. This made it possible to only identify the contribution of the performance level to BOLD variation (same method as Wolbers et al., 2007).

To assess whether one type of spatial processing involved the hippocampal formation to a greater extent, we defined an a priori ROI mask for each hippocampus (left and right) based on the anatomic definition of the hippocampi using WFU PickAtlas (http://www.nitrc.org/projects/wfu_pickatlas/, Tzourio-Mazoyer et al., 2002). The percentage of signal change was extracted using Marsbar (http://marsbar.sourceforge.net/) from each spatial condition (allocentric hits and egocentric-updated hits) and compared using a *T*-test.

## Results

The fMRI analysis first compares the cerebral activity for words correctly retrieved elicited by the spatial processes performed during learning (i.e., egocentric-updated or allocentric). Retrieval areas specifically activated by each type of spatial condition will reflect areas linking each spatial process to episodic retrieval. An a priori anatomical ROI analysis of the hippocampi was performed to investigate the differential involvement of this structure during automatic retrieval processing, following each spatial condition (allocentric and egocentric-updated). Then, we assessed which cerebral regions of the retrieval network are modulated by good episodic memory retrieval performance using a multiple regression analysis.

### Regions driven by the spatial processes performed during learning

#### Whole brain analysis

**Figure 4:**
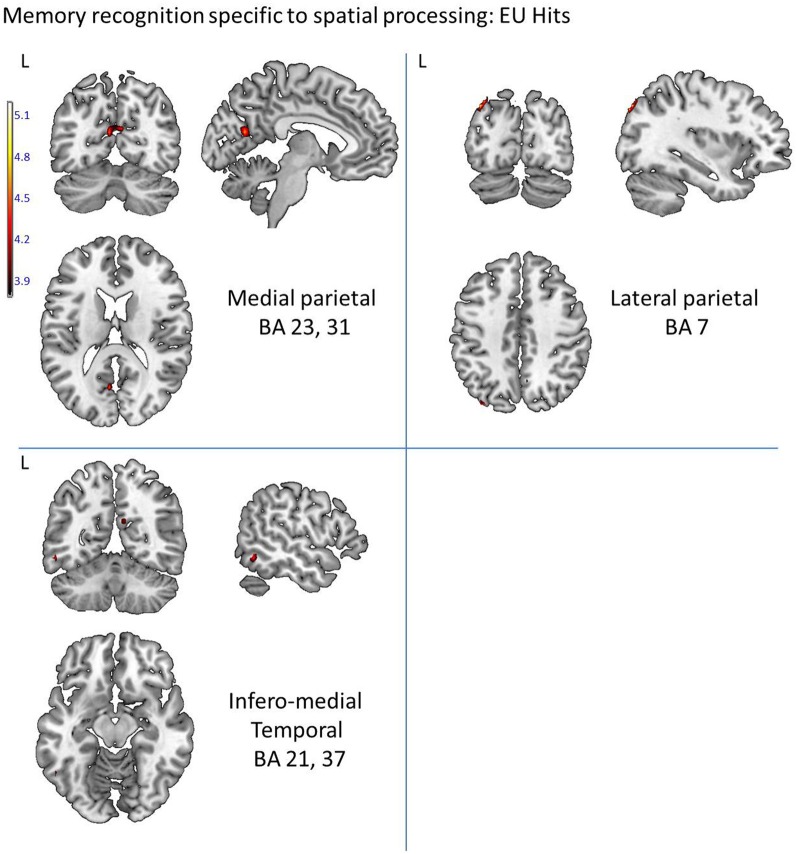
**Activated regions for word recognition after spatial training, which are modulated by the retrieval performance**. They were provided by a multiple regression (correlation) analysis with cross-subject correct detection score serving as a predictor variable, and are represented by: the left precuneus and posterior cingulate cortex (BA 23, 31) and the hippocampus. The statistical significance threshold for individual voxels was set empirically at uncorrected *p* < 0.0001. The activation was projected onto 2D anatomical slices (T1-template image) in neurological convention (Left, L is Left). Coloring indicates, for each voxel, on the upper image, the *t*-value and, on the lower image, the correlation coefficient value.

We first aimed to identify the cerebral regions modulated by the type of spatial condition during correct word retrieval (Hits). The contrast [*allocentric Hits vs. egocentric-updated Hits*] did not reveal significant activation but [*egocentric-updated Hits vs. allocentric Hits*] induced activation of a left temporo-parietal network. These regions are illustrated in Table 1 and Figure 4. They were the following: (1) the medial parietal area including the precuneus and the posterior cingulate (BA 23, 31) gyrus, with an activation peak at (−6, −63, 19) in the Talairach coordinate and extending over 53 voxels (2) the left postero-lateral parietal area including the superior parietal lobule (BA 7), with an activation peak at (−36, −77, 43) and extending over 21 voxels. This activation was rather posterior (Figure 4) and according to the functional parcellation defined by Nelson et al. (2010) it may rather corresponds to the inferior parietal lobule; (3) the left temporal area including the inferior and the middle temporal gyri (BA 37, 21) with an activation peak at (−56, −56, −6) and an extent of 14 voxels. These clusters were resistant to non-stationarity corrections (Hayasaka et al., 2004) illustrating their statistical robustness.

**Table 1:** **Activated regions for word recognition which are driven by the *egocentric-updated* spatial processing performed during the learning phase**.

**Contrast**	**Area**	**Side**	**BA**	***k***	***X*-coor**	***Y*-coor**	***Z*-coor**	***T***
Egocentric-updated specific processing [EU Rec. > A Rec.]	Precuneus, superior parietal lobule	L	7, 19	21	−36	−77	43	5.23
	Inferior temporal gyrus, middle temporal gyrus	L	21, 37	14	−56	−56	−6	4.74
	Precuneus, posterior cingulate	L	23, 31	53	−6	−63	19	4.72

They were provided by the contrast egocentric-updated Hits vs. Allocentric Hits: the left precuneus and superior parietal, and the bilateral precuneus and posterior cingulate cortex and also the left inferior and middle temporal gyri. The statistical significance threshold was set at p < 0.0001 (random-effect analysis) with a cluster extent of k ≥ 10 voxels. The Talairach coordinates (x, y, z) are indicated for each voxel. The side, Right (R) and Left (L), gyri and Brodmann areas (BA) are mentioned.

#### Anatomical ROI analysis of the retrieval hippocampal activity triggered by each spatial process performed during learning

The *T*-test revealed no significant difference between the percentage of signal change in the allocentric hit condition and in the egocentric-updated hit condition in both the left and the right hippocampi (Ts < 1).

### Memory regions

#### Whole brain analysis

The comparison between Hits vs. New words correct rejection recruited a large fronto-temporo-parietal neural network previously identified in memory recognition processing studies (Table 2). The largest cluster of activation was found in the precuneus bilaterally, (BA 7, 31). Other areas of activation included the bilateral parahippocampal gyrus (respectively on the left and right hemispheres, BA 27, 30), including the bilateral hippocampus and the right caudate nucleus. As also expected during a recognition task, the activation was also found in the left middle and superior frontal gyrus (BA 8) and extended to the post-central and pre-central gyrus (BA 3, 4, 6).

**Table 2:** **Activated regions for word recognition hits vs. Correct rejections**.

**Contrast**	**Area**	**Side**	**BA**	***k***	***X*-coor**	***Y*-coor**	***Z*-coor**	***T***
Memory recognition processing [A Rec., EU Rec. > New Rej.]	Precuneus	L	7	852	−6	−68	39	9, 4
	Parahippocampal gyrus	L	HC, 27	44	−27	−30	−7	6, 09
	Parahippocampal gyrus	R	HC, 27	26	24	−35	−1	5, 24
	Middle frontal gyrus	L	8	74	−36	22	41	4, 49
	Post-central gyrus	R	3, 4	56	48	−15	56	4, 24

The statistical significance threshold for individual voxels was set at p < 0.05 (FDR corrected). The Talairach coordinates (x, y, z) are indicated for each voxel. The side, Right (R) and Left (L), gyri, and Brodmann areas (BA) are mentioned.

#### Word recognition regions modulated by memory performance (hits) as provided by the multiple regression/correlation analysis

**Figure 5:**
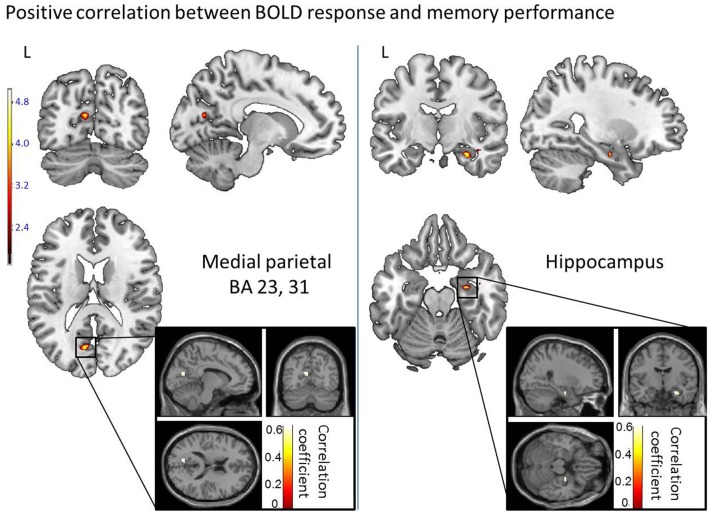
**Activated regions for word recognition which are driven by the *egocentric-updated* spatial processing performed during the learning phase**. They were provided by a random effect analysis and contrasting *egocentric-updated Hits vs. Allocentric Hits* and are represented by: the left precuneus and superior parietal (BA 7), and the bilateral precuneus and posterior cingulate cortex (BA 23, 31) and also the left inferior and middle temporal gyri (BA 21, 37). The activation was projected onto 2D anatomical slices (T1-template image) in neurological convention (Left, L is Left). The color scale represents the *t* value of activation. The statistical significance threshold set for individual voxels at uncorrected *p* < 0.0001 (random-effect analysis).

The multiple regression analysis which included for each participant the correct word detection score and the BOLD value (MR signal intensity variation) allowed the identification of the recognition regions modulated by the performance (number of correct recognitions) and distinguished regions activated by good memory recognition performers. Based on the measured correct detection scores (hits), our subjects were either lower or higher performers. The correlation analysis revealed five regions showing positive correlation between performance and BOLD response in temporo-parietal areas (with all correlation coefficients greater than 0.5, see Figure 5). In temporal areas, they were the right hippocampus (50 voxels), the left lingual and left fusiform gyri (BA 19, 37, 59 voxels). In the parietal areas, the left precuneus was activated over 15 voxels with a peak located at (−9, −69, 18) in Talairach space (BA 23, 31). The brainstem was also activated in the pons over 16 voxels (Figure 5). Compared to lower, the higher performers showed a significantly greater magnitude of activation of these regions.

## Discussion

Our study showed that, at the cerebral level, the retrieval of a word (a non-spatial element) learnt concomitantly with a task maximizing egocentric-updated spatial processing during learning was different from the retrieval of a word learnt concomitantly with a task maximizing allocentric spatial processing. The temporal and parietal areas were involved in this distinction but no hippocampal difference was found. This fMRI study is of interest for theoretical models linking episodic memory and spatial cognition (O'Keefe and Nadel, 1978). In fact, the Cognitive map theory yields that the hippocampal structure binds all the neocortical representations related to an episode during learning through a spatial scaffold. At retrieval, the hippocampus would help to index neocortical information with this allocentric spatial scaffold (O'Keefe and Nadel, 1978). Moreover, recent models of episodic memories have emphasized the involvement of the egocentric perspective in recollection (Simons et al., 2008; Gomez et al., 2009, 2012; Ciaramelli et al., 2010). Both models suggest that the retrieval of a non-spatial element from episodic memory automatically involves spatial processes. They predict that spatial processes performed during learning should influence the retrieval of a non-spatial element but they make distinct predictions on the type of spatial processes involved (egocentric-updated and allocentric). Yet, no distinction at the cerebral level of their influences during the retrieval of a non-spatial element was ever described.

We determined that learning a word while maintaining an egocentric-updated spatial information enhanced the retrieval cerebral activity and that this modulation occurred within the temporo-parietal pathway. Importantly for the conclusions to be drawn from this experiment, this distinction in the cerebral state occurred although the behavioral responses were not significantly different between egocentric-updated and allocentric conditions. Therefore, differential activity could not be attributed to activity related to a differential feeling of success across conditions. Moreover, because no difference was observed in the proportion of detailed vs. simple recognition across the two spatial conditions, the differential activity is not simply related to a difference in terms of level of retrieval.

Importantly, a close inspection of the modulation of cerebral activity within the hippocampal formation did not allow observation of a significant difference between retrieving information learnt in an allocentric condition from information learnt in the egocentric-updated condition. Further replications will be necessary to provide clear-cut conclusions on this observation. However, if this result is confirmed, no discrepancy would appear with models linking episodic and allocentric spatial memory. For instance, the memory of the egocentric-updated process (Gomez et al., 2009) added to the BBB model, can be seen as an epiphenomenon (giving rise to a fluency mechanism) which does not interfere with (i.e., enhance or decrease) the binding mechanism of the hippocampal region per se (for a review, see Konkel et al., 2009). This binding mechanism could be important in both spatial conditions (egocentric-updated and allocentric) as suggested by the correlation of the BOLD response in this region with the memory performance of participants. In fact, the correlational analysis of memory performance in this study did involve a right hippocampal cluster. This correlation of the right hippocampal activity with the level of memory performance is in agreement with previous memory studies (Gabrieli et al., 1997; Eichenbaum et al., 2007). Such modulation of the right hippocampal region is coherent with the binding mechanism of the models linking episodic memory and spatial processing. According to these models, the binding mechanism led by the hippocampal formation would be critical in linking the content of the memory (stored in the perirhinal regions) to its spatial attributes (Brown and Aggleton, 2001).

Most importantly, the experiment shows that the egocentric-updated spatial processing performed during learning enhances retrieval activity within the temporo-parietal pathway, as predicted by the BBB model (e.g., Burgess et al., 2001b) and the Gomez et al. (2009) hypothesis. The differential substrate observed in the egocentric-updated condition needs to be puzzled out. Based on the theoretical framing of the experiment and on reverse inference (Poldrack, 2006, 2011), “mind-reading” of the mental state suggests that the most plausible interpretation of the cognitive mechanisms reflected by the temporo-parietal activity could be self-projection in space and time. The implication of each of the three following regions will now be discussed: (1) the medial parietal (precuneus and posterior cingulate) region, (2) the lateral parietal region, and (3) the infero-medial temporal region.

### The medial parietal region

The medial parietal region is a crucial component of the Default Mode Network (DMN) (Buckner et al., 2008; Spreng and Grady, 2009; Spreng et al., 2009), highly activated during conscious resting state and deactivated during cognitive tasks (Shulman et al., 1997; Mazoyer et al., 2001; Raichle et al., 2001; Buckner et al., 2008; Spreng and Grady, 2009; Spreng et al., 2009). More broadly, co-activation of DMN regions in a wide variety of processes, such as retrieval of autobiographical memory, prospection, spatial navigation and theory of mind, led researchers to believe that these structures belong to a “*core network*” (Spreng and Grady, 2009; Spreng et al., 2009). The “*core network*” would support the common aspects of many cognitive behaviors and mechanisms and would reflect the simulation of internalized experience, as well as self-projection (Buckner and Carroll, 2007; Tsakiris et al., 2010). Hence, in the present study, the activation of DMN-like regions in the recognition task, after learning the word while performing an egocentric-updated processing, may reflect the simulation of internalized experiences, as well as self-projection that are particularly involved in recollection.

In fact, the precuneus extending to posterior cingulate and retrosplenial cortices is also known to be decisive in episodic memory function (Rugg et al., 2002; Shannon and JBuckner, 2004; Naghavi and Nyberg, 2005; Wagner et al., 2005; Cavanna and Trimble, 2006; Cabeza, 2008; Cabeza et al., 2008; Hutchinson et al., 2009; Uncapher and Wagner, 2009; Uncapher et al., 2010). The precuneus has been involved in numerous studies of episodic memory retrieval (Lundstrom et al., 2003, 2005; Addis and Tippett, 2004; Addis et al., 2004; Gilboa et al., 2004; Viard et al., 2010, 2011), self-processing (Kircher et al., 2000, 2002; Ruby and Decety, 2001; Vogeley et al., 2001; Lou et al., 2004) and visuo-spatial imagery such as mental navigation, mental rotation and motor imagery (Ghaem et al., 1997; Malouin et al., 2003). Moreover, assessing correlations between BOLD response in the correct retrieval network and the level of memory performance revealed that a parietal region also located in the precuneus was more active in high memory performers. This correlational result suggests that, beyond the fact that egocentric-updated hits activate a supplementary area in regions devoted to memory mechanisms, it does so in regions which are related to good memory performance. This overlap of parietal activation from the correlational analysis and the main contrast suggest that the activity observed in the egocentric-updated hits vs. allocentric hits contrast might reflect a mechanism critical to memory retrieval.

Since medial parietal regions seem to be particularly involved in episodic retrieval, the greater retrieval activity in the egocentric-updated condition suggests that this spatial process is a key component of episodic memory. One possible interpretation is that it could provide a spatial mechanism to simulate internalized experience and self-projection. In such a case, it could also provide a spatial mechanism to simulate internalized experience and self-projection when participants rest, triggering the observed greater activity of this region in the so-called DMN.

### The lateral parietal region

Next, the activity of the posterior part of the left lateral parietal lobule was also enhanced when retrieval concerned the items learnt under the egocentric-updated condition. Such differences in terms of visual imagery have been shown to involve the left posterior parietal activity This activity has been previously correlated to recollection effects such as the perceived oldness effect, the recollection vs. familiarity distinction and the retrieval orientation effect (i.e., Source-Item). Four alternative hypotheses have been considered to account for these effects (Wagner et al., 2005): (1) the mnemonic accumulator hypothesis, (2) the output buffer hypothesis, (3) the attention to internal representation hypothesis, and (4) the subjective memory hypothesis.

- *The mnemonic accumulator hypothesis* proposes that parietal regions temporally integrate a memory-strength signal, thus contributing to decision criteria to the eventual decision. In this view, the activity of the left lateral parietal areas in the egocentric updated condition would reflect a memory strength signal difference.
- *The output buffer hypothesis* posits, in line with the Baddeley's working memory buffers, that parietal regions “hold” retrieved information in a form, accessible to decision-making processes. In this view, the activity observed in the spatial processing contrast of our study would reflect the reinstantiation of more visuo-spatial sensory-information, or more imagery.
- *The attention to internal representation hypothesis* and *the dual attentional processes hypothesis* (also called the *attention to memory model*) (Cabeza et al., 2003, 2008; Cabeza, 2008), proposes that the posterior parietal cortex might contribute to shift attention to, or maintain attention on, internally generated mnemonic representations—perhaps those dependent on the medial-temporal lobe. However, recent reviews (Hutchinson et al., 2009; Uncapher et al., 2010) and connectivity results (Nelson et al., 2010; Uncapher et al., 2010) challenge this view, indicating that the correspondence between attention and episodic retrieval effects in posterior parietal cortex seems more apparent than real. Given the activation peak of our data, in the *dual attentional hypothesi*s, this activity would not reflect effortful memory decision but rather reflect exogenous attention due to stimulus-driven saliency effects.
- *The subjective memory hypothesis* proposes that the parietal lobe is responsible for the subjective experience of confidence and vividness in one's retrieved memories, the access to subjective states of awareness (Ally et al., 2008). More specifically, the parietal lobe is related to cognitive functions engaging the individual in a higher degree of self-relevant information processing (e.g., meditational state, out-of-body-experience (Lou et al., 1999, 2005; Kjaer et al., 2002; Blanke et al., 2008; Blanke and Metzinger, 2009). Recently, it has been revealed that this region would be crucial for episodic memory encoding, and could impact subjective recollective experience, throughout mental imagery [i.e., autonoetic consciousness, (Wagner et al., 2005; Berryhill et al., 2007; Ally et al., 2008; Vilberg and Rugg, 2008; Olson and Berryhill, 2009; Simons et al., 2009)]. Several neuroimaging evidence (Chua et al., 2006; Duarte et al., 2008), as well as parietal patients statements (Ally et al., 2008; Davidson et al., 2008) or neuropsychological evaluations showing decreased levels of memory vividness or confidence (Berryhill et al., 2007, 2010; Simons et al., 2008, 2009; Drowos et al., 2010) support this hypothesis. This last view appears coherent with the interpretation of the medial parietal activation suggesting that egocentric updating is a key component of the episodic memory probably by providing a self-referential system across time.

Finally, this region is more active when participants simulate the movement of an object in 3D space compared to 2D space (e.g., Kawamichi et al., 2007). In our experiment, this activity might also reflect a more immersive 3D space re-experiencing of the event in the egocentric-updated condition compared to a non-immersive 2D space retrieval from the allocentric condition.

In summary, the exact signification of the left lateral parietal region modulation by the spatial processes performed during learning remains unresolved as this retrieval activity in the egocentric-updated condition could reflect (1) a greater memory strength signal, (2) the reinstantiation of more visuo-spatial sensory-information (3) an exogenous attentional difference, due to a stimulus-driven saliency, (4) more likely an enhancement of self-referential processing across time, and lastly, (5) a difference in the spatial re-experiencing. However, independently of its interpretation, the implication of both the left lateral parietal and the medial parietal region supports the hypothesis that processing egocentric-updated during item encoding might influence its subsequent neuronal retrieval by modulating areas known to support recollection.

### Infero-medial temporal regions

The third region activated was the inferior and middle temporal area, which could be related to top-down visual working memory that directs the mind's eye. In line with the literature on working memory for visual objects, we suggest that this activation might reflect the manipulation of visual images through top-down processes. These regions are implicated in the ability to recall, maintain and manipulate visual images in the absence of external stimulation (Ranganath et al., 2004, 2005; Ranganath and D'Esposito, 2005; Ranganath, 2006). We propose that a concomitant egocentric-updated spatial task while learning a word might have facilitated reliving the whole event through mental imagery during word retrieval.

## Conclusion

Our results revealed that enhancing egocentric-updated processing during word learning increases the activation of medial and lateral parietal and temporal regions during retrieval even if the recognition question focuses on the words themselves. Various cognitive mechanisms may explain the described modulation of the temporo-parietal regions (i.e., subjectivity, attention to memory, self-projection in space and time…). Although conclusions must await further investigations, currently only the BBB model and in particular the Gomez et al. (2009) hypothesis can account for such results. In fact, the BBB model posits an involvement of the parieto-temporal pathway in the egocentric-updated processing. The involvement of the parieto-temporal connections in episodic memory retrieval related to the egocentric-updated spatial process performed during learning can provide an interesting link to the reduction in temporo-parietal gray matter volume of amnesic patients (Vargha-Khadem et al., 2003; Salat et al., 2006).

### Conflict of interest statement

The authors declare that the research was conducted in the absence of any commercial or financial relationships that could be construed as a potential conflict of interest.
